# Comparison of Heart Rate Variability in People With Diabetes-Related Neuropathic Foot to Their Counterparts Without a Foot Ulcer History: A Propensity Score Matching Study

**DOI:** 10.1155/jdr/3349391

**Published:** 2025-05-30

**Authors:** Murong Wu, Shuang Lin, Yan Liu, Dawei Chen, Xingwu Ran, Chun Wang, Lihong Chen, Sen He, Donge Yan, Mingxin Bai, Yingying Dong, Wen Wang, Zhiyi Lei, Yun Gao

**Affiliations:** ^1^Diabetic Foot Care Center, Department of Endocrinology and Metabolism, West China Hospital, Sichuan University, Chengdu, Sichuan, China; ^2^Department of Endocrinology, Chengdu Eighth People's Hospital, Chengdu, Sichuan, China; ^3^Department of Cardiology, West China Hospital, Sichuan University, Chengdu, Sichuan, China; ^4^Department of Endocrinology and Metabolism, Central Hospital of Dalian University of Technology, Dalian, Liaoning, China; ^5^Chinese Evidence-Based Medicine Center, West China Hospital, Sichuan University, Chengdu, Sichuan, China; ^6^West China Medical School, Sichuan University, Chengdu, Sichuan, China

**Keywords:** cardiac autonomic function, diabetic foot ulceration, heart rate variability, propensity score matching

## Abstract

**Background:** The reasons that individuals with diabetic foot ulceration (DFU) have higher cardiovascular mortality than those with Type 2 diabetes mellitus (T2DM) but without DFU remain controversial. We aimed to compare the differences in cardiac autonomic function between individuals with neuropathic DFU and their counterparts without DFU.

**Methods:** Three hundred and sixty-two participants with T2DM (181 with DFU and 181 without DFU) who were free of peripheral artery disease (PAD) were included in the final analysis after propensity score matching (PSM). All individuals underwent a 24-h ECG Holter and used the following indices of heart rate variability (HRV) to assess cardiac autonomic function: the standard deviation of the normal sinus interval (SDNN), the root mean square of successive RR interval differences (rMSSD), the standard deviation of the 5-min average RR intervals (SDANN), the percentage of normal adjacent RR interval difference > 50 ms (PNN50), the low-frequency power (LF), the high-frequency power (HF), and the LF/HF ratio.

**Results:** Individuals with DFU had lower SDNN, LF/HF, PNN50, rMSSD, HF, SDANN, and LF than their counterparts without DFU (all *p* < 0.05). Individuals with DFU had a 2.5-fold increase in severe impairment of cardiac autonomic modulation (i.e., SDNN < 50 ms) compared to those without DFU (21.6% vs. 8.8%, *p* < 0.001). DFU was independently and negatively associated with all the abovementioned HRV measures (all *p* < 0.05).

**Conclusion:** Among people with neuropathic diabetic foot only, cardiac autonomic function was still more severely impaired in individuals with DFU than in their counterparts without DFU.

**Trial Registration:** CHiCTR2300076628

## 1. Introduction

Diabetic foot ulceration (DFU), which usually develops in a person with diabetes mellitus (DM) simultaneously having diabetes-related peripheral neuropathy and/or peripheral artery disease (PAD) in the lower limbs, is one of the most serious late complications of DM [1]. The incidence of DFU has been rising with the worldwide prevalence of DM and the increased longevity of people with DM [2].

Although both individuals with DM with and without DFU are at increased risk of cardiovascular and all-cause deaths due to various diabetic complications or comorbidities, previous studies have shown that individuals with DFU have a higher mortality rate than those without DFU, mainly due to cardiovascular diseases [3–7]. The risk of cardiovascular mortality in individuals with DFU is approximately three times higher than that of those without DFU [4, 8]. Thus, considering the same disease context of DM, the reasons for this mortality discrepancy have attracted a certain attention.

The causes of diabetes-related cardiovascular death have primarily focused on coronary atherosclerotic heart disease and ischemic cerebrovascular disease, which were more prevalent among patients with DFU than among those with diabetes and no history of foot ulcers. Due to the presence of ischemic foot, patients with DFU usually have a higher prevalence of coronary artery disease (CAD) [9], which could explain the abovementioned mortality discrepancy.

Actually, apart from the adverse condition for developing CAD, persistent hyperglycemia-mediated pathophysiological alteration and metabolic distortion also lead to the impairment of cardiac autonomic function via other mechanistic pathways independent of atherosclerosis and myocardial ischemia [10, 11]. In our previous study [12], we investigated the differences in cardiac autonomic function between individuals with DFU and those with diabetes but without DFU. The results showed that the indices of heart rate variability (HRV) were significantly lower in patients with DFU compared to their counterparts without DFU, indicating more severe impairment of cardiac autonomic function in individuals with DFU. However, the study did not exclude cases of ischemic or neuroischemic foot, which may be associated with a higher incidence of CAD in individuals with DFU, potentially affecting cardiac autonomic function and thereby influencing the interpretation of the study's findings. Thus, if the interference of neuroischemic or ischemic foot is excluded, do individuals with neuropathic foot still exhibit distinct changes in cardiac autonomic modulation compared to those with DM but without a history of foot ulcers?

Currently, HRV is the most sensitive indicator for assessing the cardiac autonomic nervous system; decreased HRV has predictive value for adverse cardiovascular events and mortality among people with DM [13, 14]. The relevant indicators of HRV can be obtained through 24-h ECG Holter, which has the advantages of being simple and noninvasive. Thus, we employed HRV analysis to evaluate whether people with neuropathic foot have different alterations in cardiac autonomic modulation compared to their counterparts with Type 2 diabetes mellitus (T2DM) but without DFU.

## 2. Materials and Methods

### 2.1. Study Population

A total of 2944 individuals with T2DM with or without DFU were recruited from the Diabetic Foot Care Center of West China Hospital, Sichuan University, between January 2016 and June 2023. We collected information on demographic features, lifestyle risk factors, history of comorbidities, and measured biochemical indices (e.g., glycosylated hemoglobin (HbA1c), glucose, and lipid) in the fasting state. DM was diagnosed according to the World Health Organization (WHO) report (1999) [15]. DFU was defined based on the criteria of the International Working Group on the Diabetic Foot (IWGDF) [1]. LVEF was calculated by Simpson's method and obtained from echocardiography [16]. PAD was identified through a history of revascularization therapy or symptoms and signs such as claudication, absent pulses, or examinations such as ankle–brachial index (ABI), the lower-extremity color Doppler ultrasound, and angiography [17, 18]. The value of ABI ≤ 0.9, a greater than 50% diameter reducing stenosis or occluded arteries were considered for PAD.

Exclusion criteria were as follows: incomplete medical records (e.g., 24-h Holter ECG), PAD, LVEF < 50%, eGFR < 30 mL/min/1.73m^2^, special types of diabetes or other endocrine diseases, other lower-extremity ulcers (e.g., venous ulcers, pressure ulcers), severe hepatic or renal insufficiency, severe cardiovascular diseases such as myocardial infarction and arrhythmias, chronic obstructive pulmonary disease, respiratory failure, severe infections, hematologic diseases, rheumatologic diseases, malignancy, hemorrhage, Parkinson's syndrome, and treatment with medications affecting HRV.

### 2.2. 24-h Holter ECG

A 24-h Holter ECG system (GE-Marquette MARS PC) was employed for 24-h ECG monitoring. The procedure was performed by trained professionals at the ECG unit of West China Hospital of Sichuan University. The operator should inform the participants about the procedure, device, and precautions. Participants should maintain a regular sleep schedule, abstain from stimulants (e.g., alcohol, narcotics, and coffee), and limit intense physical exercise while continuing normal daily activities for at least 48 h. Each participant underwent a 24-h Holter ECG at the same time (8–9 a.m.) to avoid bias from circadian rhythms. Electrode pads were positioned in the precordial region where the skin should be kept dry for accurate recording of ECG activity. Participants should also be electrified away from electromagnetic radiation during recording to avoid interference with ECG signals.

### 2.3. HRV Measures

The recorded ECG signal was processed by a Holter Analysis Workstation (GE Medical Systems Information Technologies Inc., Texas, United States). R-peaks were identified using an automatic peak-finding procedure and manual editing to estimate RR intervals. After RR intervals were calculated, abnormal heartbeats and artifacts were excluded from ECG recordings to achieve more reliable results. After preprocessing, the time-domain indices of HRV including the standard deviation of the normal sinus interval (SDNN), the root mean square of successive RR interval differences (rMSSD), the standard deviation of the 5-min average RR intervals (SDANN), and the percentage of normal adjacent RR interval difference > 50 ms (PNN50) were generated. Next, a discrete Fourier transform of the RR time series based on the Welch method was performed to calculate the frequency domain indices of HRV including the low-frequency power (LF), high-frequency power (HF), and LF/HF ratio.

In the above indicators, SDNN < 50 ms for highly depressed HRV, and SDNN ≥ 50 ms but < 100 ms for moderately depressed HRV, both of which indicate impairment of cardiac autonomic modulation [14, 19].

### 2.4. Assessment of Covariates

We collected information on covariates including diabetic duration, current smoking, alcohol consumption, family history of DM, comorbidities, and complications of DM (e.g., hypertension (HBP), ischemic cerebrovascular disease (CVD), diabetic peripheral neuropathy (DPN), CAD, and PAD). HBP is defined as systolic blood pressure (SBP) values ≥ 140 mmHg and/or diastolic blood pressure (DBP) values ≥ 90 mmHg [20]. The history of CAD and CVD was ascertained based on reviewing medical records. DPN was defined as the presence of symptoms and two or more signs or the presence of abnormal nerve conduction in people with diabetes after the exclusion of other causes. The symptoms included pain, dysesthesia, burning, tingling, and numbness. The 10-g monofilament, 128-Hz tuning, pin and Tip-Therm and ankle reflexes were used to evaluate neuropathy signs. Additionally, all patients underwent electromyography tests to assess nerve conduction [21–23]. Based on the standard from the Center for Neurobiological Detection at West China Hospital of Sichuan University, abnormal nerve conduction studies (NCSs) for sensory nerves of the upper limbs, such as the ulnar and median nerves, were defined as conduction velocities less than 50 m/s or amplitudes less than 8 *μ*V. For motor nerves of the upper limbs, such as the ulnar and median nerves, abnormal NCSs were defined as conduction velocities less than 50 m/s or amplitudes less than 8 mV. For sensory nerves of the lower limbs, such as the sural nerve, abnormal NCSs were defined as conduction velocities less than 50 m/s or amplitudes less than 10 *μ*V. For motor nerves of the lower limbs, such as the tibial and common peroneal nerves, abnormal NCSs were defined as conduction velocities less than 45 m/s or amplitudes less than 3 mV. Body mass index (BMI) was calculated as weight (kilogram) divided by height squared (square meters) [24]. The concentrations of fasting plasma glucose (FPG), glycated hemoglobin (HbA1c), triglycerides (TG), high-density lipoprotein cholesterol (HDL-C), low-density lipoprotein cholesterol (LDL-C), and serum creatinine were determined by the central laboratory of West China Hospital. Estimated glomerular filtration rate (eGFR) was calculated after modification of serum creatinine, sex, and age.

### 2.5. Statistical Analysis

Statistical analysis was performed using SAS 9.4 software (SAS Institute Inc., Cary, North Carolina). Continuous variables were expressed as mean ± SD or median and range as appropriate, while categorical variables were expressed as frequencies or percentages. Continuous variables between the two groups were compared by Student's *t*-test or Wilcoxon's rank-sum test as appropriate. A *χ*^2^ test and Fisher's exact test were used for categorical data as appropriate. We employed propensity score matching (PSM) among participants to minimize the potential imbalance of confounding factors between groups. With the use of multivariable logistic regression, baseline characteristics that were statistically significant (*p* < 0.05) and/or considered potential confounders were included, and the propensity scores (PSs) were calculated. Finally, age, sex, diabetic duration, BMI, mean blood pressure (MBP), alcohol consumption, current smoking, family history of DM, HBP, CAD, CVD, DPN, eGFR, TC, TG, and HbA1C were included in the model, and 1:1 matching method was used with the nearest neighbor caliper width of 0.05. To analyze covariate differences before and after PSM, the absolute standardized differences (ASDs) for each covariate (an ASD ≥ 0.10 indicates imbalance) were calculated. The MatchIt package for R (RStudio, PBC, Boston, Massachusetts, United States) was used to accomplish this matching procedure. Additionally, univariate and multivariate regression analyses were also carried out after PSM to evaluate the relationship between DFU and variables related to cardiac autonomic function. A two-sided *p* value of 0.050 was regarded as statistically significant for all statistical analyses.

## 3. Results

**Figure 1:**
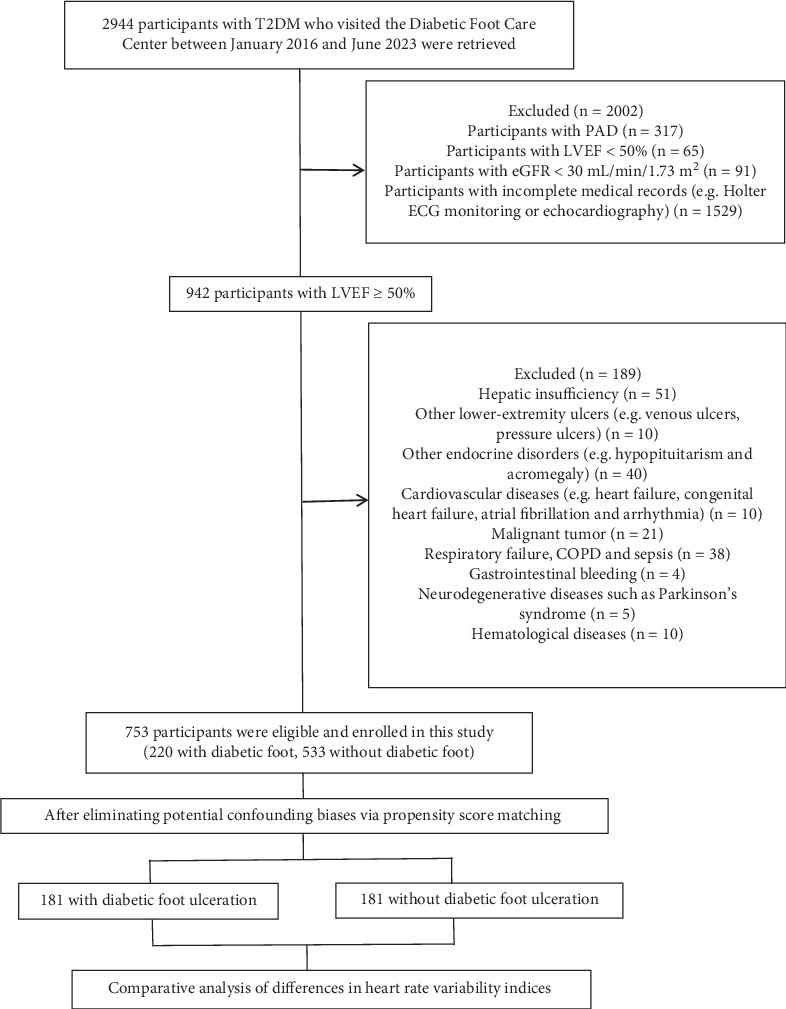
Flowchart of the study selection process. Abbreviations: T2DM, Type 2 diabetes mellitus; PAD, peripheral arterial disease; LVEF, left ventricular ejection fraction; eGFR, estimated glomerular filtration rate; COPD, chronic obstructive pulmonary disease.

The study selection process is shown in Figure 1. Based on exclusion criteria, participants with PAD were excluded, and 753 patients (220 with DFU and 533 without DFU) were included. In order to minimize the potential imbalance of confounding factors between the two groups, we then employed PSM among the participants. Consequently, a total of 362 participants with T2DM (181 with DFU and 181 without DFU) were ultimately enrolled in the study. Among the 181 participants with DFU, 116 had active DFU while 65 had healed DFU.

**Figure 2:**
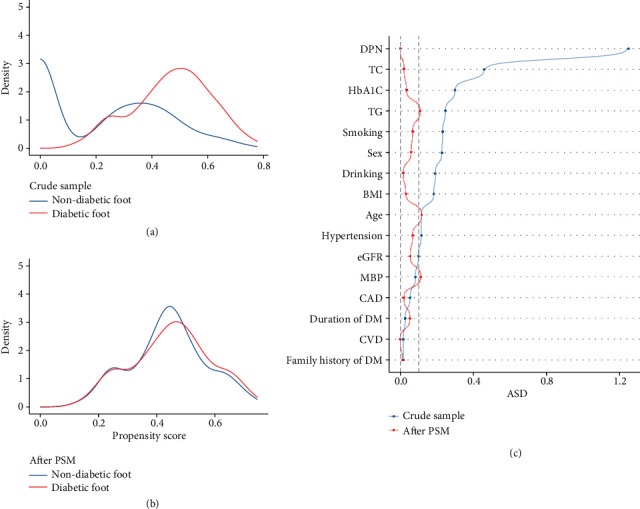
Propensity score distributional overlap and absolute standardized differences in participants with diabetic foot ulceration and without diabetic foot ulceration. (a, b) Propensity score (PS) distributions between individuals with or without DFU before and after PSM. Greater overlap of PS curves of the two groups indicates a lesser risk of confounding. (c) Dot plot of absolute standardized differences before and after matching. The dashed line indicates greater than 0.1 imbalance between the variable's value. Abbreviations: ASD, absolute standardized difference; PSM, propensity score matching; DM, diabetes mellitus; CVD, cerebrovascular disease; CAD, coronary artery disease; MBP, mean blood pressure; eGFR, estimated glomerular filtration rate; BMI, body mass index; TG, triglycerides; TC, total cholesterol; HbA1c, glycated hemoglobin; DPN, diabetic peripheral neuropathy.

Figure 2a,b shows the distribution of PS in participants with and without DFU before and after matching, demonstrating the adequateness of PSM and the well-balanced baseline covariates between the two groups after PSM.  Figure 2c shows the ASD before and after PSM. Several unbalanced variables (including TG, age, and MBP) with ASD ≥ 0.1 were further adjusted in the multivariate analysis.

The demographic and clinical characteristics of all participants after PSM are shown in Table 1. Individuals with DFU exhibited higher levels of SBP and lower levels of HDL-C; however, no significant differences were observed between the two groups in terms of sex, age, diabetic duration, prevalence of drinking and smoking, BMI, DBP, HbA1c, FPG, TC, TG, LDL-C, eGFR, and the proportions of DPN, HBP, CAD, and CVD.

Within the indices of HRV, SDNN and the LF/HF reflect the overall cardiac autonomic regulation and serve to assess the overall damage and recovery of the cardiac autonomic nerve. Individuals with DFU had lower SDNN and LF/HF than their counterparts without DFU, and the differences were statistically significant (all *p* < 0.05). Additionally, PNN50, rMSSD, and HF were used to assess cardiac vagal activity, while SDANN and LF were utilized to evaluate cardiac sympathetic activity. All of these measures were significantly lower in participants with DFU than in those with T2DM who had no history of DFU (all *p* < 0.05) (Table 2). In the univariate linear regression analysis, DFU was independently negatively associated with all the abovementioned HRV measures: SDNN (*β* −0.334; 95% CI [−0.418, −0.251]; *p* < 0.001), LF/HF (*β* −0.178; 95% CI [−0.219, −0.137]; *p* < 0.001), SDANN (*β* −0.321; 95% CI [−0.404, −0.238]; *p* < 0.001), LF (*β* −0.650; 95% CI [−0.794, −0.506]; *p* < 0.001), PNN50 (*β* −0.238; 95% CI [−0.421, −0.056]; *p* = 0.011), rMSSD (*β* −0.220; 95% CI [−0.316, −0.124]; *p* < 0.001), and HF (*β* −0.302; 95% CI [−0.413, −0.191]; *p* < 0.001). Similarly, in the multivariate linear regression, DFU continued to show an independently negative association with each HRV index (all *p* < 0.05) (Table 3).

**Figure 3:**
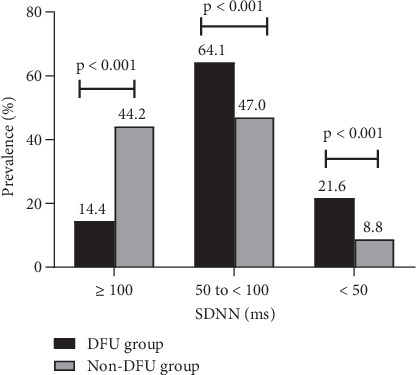
The prevalence and severity of cardiac autonomic modulation impairment in participants with and without diabetic foot ulceration. Abbreviations: DFU, diabetic foot ulceration; SDNN, the standard deviation of normal sinus interval.

SDNN is used to grade the severity of cardiac autonomic modulation impairment in patients with DM. SDNN < 100 ms, indicating impairment of cardiac autonomic modulation, was more prevalent in the DFU group than in the non-DFU group (85.6% vs. 55.8%, *p* < 0.001). In particular, participants with DFU had a 2.5-fold increase in severe impairment of cardiac autonomic modulation (i.e., SDNN < 50 ms) than their counterparts (21.6% vs. 8.8%, *p* < 0.001) (Figure 3). Furthermore, DFU was significantly associated with higher odds of cardiac autonomic modulation impairment (OR 5.170; 95% CI [3.026, 8.836]; *p* < 0.001) (Table 4).

## 4. Discussion

In the present study, we performed a rigorous screening in a large sample database based on the inclusion and exclusion criteria and then employed multiple statistical methods such as PSM to eliminate selection bias and achieve a balance of confounding factors between participants with neuropathic DFU and those with T2DM without DFU. After strict matching, the results showed participants with neuropathic DFU still had lower HRV indices including SDNN, LF/HF, SDANN, LF, PNN50, rMSSD, and HF than those counterparts with T2DM who did not develop foot ulceration, suggesting the former had more severe impairment of cardiac autonomic modulation than the latter, even if both groups had similar diabetic duration and DPN.

According to the pathologies of foot ulcers among people with DM, DFU can be classified into two groups: neuropathic foot with palpable pulses and ischemic foot without pulses, including neuroischemic foot. Compared with those with neuropathic DFU or those with DM but without DFU, individuals with ischemic foot usually have a higher prevalence of CAD [4, 6, 25–27], which undoubtedly results in more abnormalities in cardiac autonomic modulation. A question then arises as to whether there are obvious differences in cardiac autonomic modulation between individuals with neuropathic DFU and their counterparts with DM but without DFU. A cross-sectional study that included 63 patients with DFU and 30 patients with DM but without a history of foot ulcers in an Italian population reported a higher degree of activation of the parasympathetic system, expressed by the increase in HF values and a lower LF/HF ratio, in patients with DFU [28]. Considering its small sample size, the presence of ischemic foot, and various unbalanced confounding factors, it was not yet clear whether the alteration of HRV was different between patients with neuropathic DFU and their counterparts with DM who did not develop foot ulceration. In a previous prospective study from Korea that included 595 individuals with T2DM without DFU, it was found that cardiovascular autonomic neuropathy (the diagnostic criterion for which is based on cardiovascular autonomic reflex tests (CARTs)) was associated with a 4.5-fold increased risk of DFU compared to individuals with normal autonomic function [29]. However, this study did not exclude PAD, which is a major cause of ischemic/neuroischemic DFU, and there are various unbalanced confounding factors. Furthermore, the study used CARTs to diagnose CAN. In comparison to CARTs, changes in the indices of HRV can provide early information on impaired cardiac autonomic modulation, occurring prior to the diagnosis of CAN based on CARTs. The HRV index may be useful for predicting the development and adverse outcomes of CAN.

In the present study, we first excluded those participants with PAD to avoid the interference of a higher proportion of CAD in people with ischemic foot. Moreover, we also excluded the participants with LVEF < 50%, who possibly had ischemic myocardial damage. Additionally, we employed PSM to achieve a balance of potential confounding factors between participants with and without neuropathic DFU. After comparison of HRV indices between the two groups under the condition of rigorous screening, the results still showed that HRV indices, including SDNN, SDANN, PNN50, rMSSD, HF, LF, and LF/HF, of individuals with neuropathic DFU were significantly lower than their counterparts without DFU. Moreover, multivariate analysis showed an independent negative correlation between neuropathic DFU and the abovementioned indices of HRV. Thus, the present study suggests that people with DM who are prone to developing neuropathic DFU are more likely to have relatively severe abnormalities of cardiac autonomic modulation, even with similar age, diabetic duration, and DPN occurrence. However, different indices of HRV reflect different variations of cardiac autonomic modulation, including sympathovagal balance (i.e., the total power), vagal activity, and sympathetic activity. Then, another question arises as to which one of cardiac autonomic modulation was more severely impaired in individuals with DFU than in their counterparts without DFU?

HRV is a generally recognized quantitative marker of cardiac autonomic activity [14]. Within the indices of HRV, SDNN and the LF/HF ratio, which reflect the total variance of HRV, are considered as the markers of sympathovagal balance. Vagal activity is the major contributor to PNN50, rMSSD, and the HF component while sympathetic activity can be reflected by SDANN and the LF component [30, 31].

Prior studies have reported that individuals with DFU had significantly lower levels of SDNN and LF/HF ratio than those without DFU [31–34]. Similarly, our study showed a significantly lower SDNN and LF/HF ratio in participants with neuropathic DFU than in those without DFU. Therefore, compared to those with DM who did not develop foot ulceration, people with neuropathic DFU have greater cardiac sympathovagal imbalance. Additionally, based on the standards of physiological interpretation and clinical use of HRV, a diminished HF reflects a decrease in vagal activity directed to the heart [30, 31]. Tuttolomondo et al. reported that individuals with DFU were negatively correlated with HF and rMSSD [28]. In the present study, HF, rMSSD, and PNN50 were not only significantly lower in participants with neuropathic DFU but also were independently negatively associated with neuropathic DFU. Therefore, these studies support that people with DFU have more severely impaired vagal modulation and lower parasympathetic activity than their counterparts with DM but without DFU. It is more likely to lead to cardiac electrical instability among people with DFU, which undoubtedly amplifies the risk of cardiovascular events or malignant arrhythmia.

In terms of sympathetic modulation, Ahmed et al. reported that participants with diabetic neuropathic ulcers had significantly impaired autonomic function compared to those with DM who have never had a foot ulceration, in which the vagal nervous system was impaired earlier and more severely affected than sympathetic function [35]. In our study, we observed significantly lower SDANN and LF in participants with neuropathic DFU than in those without DFU, which suggested that cardiac sympathetic function was more severely impaired in individuals with neuropathic DFU. However, it is less clear whether impaired sympathetic function presents a relative sympathetic overdrive due to reduced vagal activity or a significant decrease in sympathetic activity, which will be observed in our further study.

Few studies have analyzed the reasons for the significant differences in cardiac autonomic dysfunction between patients with neuropathic foot disease and those with diabetes but without DFU. However, an analysis from the perspective of the pathogenesis of diabetic cardiac autonomic neuropathy (DCAN) may provide an indirect insight into the underlying factors. To our knowledge, multiple relevant mechanisms have been implicated in the pathogenic process: (1) hyperglycemia leads to sorbitol accumulation through activation of the polyol pathway, which in turn causes neuronal damage, ischemia, and hypoxia [36]. (2) Enhanced oxidative stress and increased oxygen free radicals induced by hyperglycemia may result in damage to vascular endothelium and neurons [37]. Endothelial dysfunction and reduced sympathetic nerve activity are strongly associated with the development of diabetic foot ulcers. (3) Formation of advanced glycation end products (AGEs) and activation of protein kinase C reduce the neuronal blood supply through thickening the basement membrane of the neurotrophic vessels [36]. (4) Excess levels of fatty acids induced by hyperinsulinism can promote specific inflammation of adipocytes, contributing to vasoconstriction and excessive production of inflammatory mediators such as IL-6, IL-8, and TNF-*α*, which are significantly associated with a decrease in HRV indices [38–41]. Previous studies have shown that people with DFU presented significantly elevated levels of AGEs and free radical production [42, 43] and a higher degree of endothelial function impairment [28] than those with DM but without a history of DFU. Thus, more abnormal pathological processes may predispose people with diabetic neuropathic foot disease to have more severe DCAN than patients with diabetes but without DFU.

Since changes in indicators of HRV precede the diagnosis of CAN based on the CARTs [44], we recommend that patients with DFU undergo 24-h ECG Holter monitoring to early assess whether there is impairment in cardiac autonomic modulation. Furthermore, it is advised that patients with impaired cardiac autonomic modulation undergo further CARTs to confirm the presence of CAN, facilitating early screening, diagnosis, and treatment. For patients with reduced HRV who do not meet the diagnostic criteria for CAN, we recommend strict control of related risk factors, including blood glucose, blood pressure, and blood lipids, as well as improving unhealthy lifestyle habits and adhering to standardized management of diabetic foot.

Strengths of our study included a relatively larger sample size of participants with DFU than previous studies, which established a good prerequisite to achieve a strict match for multilevel confounding factors between the two groups via PSM after excluding neuroischemic and ischemic DFU. Such strict control or elimination of confounding factors, especially PAD, DPN, diabetic duration, and age, has not been achieved in previous studies.

Our study has several potential limitations. First, it was designed to reveal the correlation between DFU and indices of HRV. Thus, it was impossible to infer a causal relation between key study variables. Further longitudinal studies should be conducted to investigate the temporal relation between DFU and indices of HRV. Second, the use of LF as a marker of sympathetic modulation has not reached universal agreement and remains highly controversial. LF is considered by some to be a marker of sympathetic modulation, especially when expressed in normalized units, while others view LF as a parameter that includes both sympathetic and vagal influences [14]. Therefore, conclusions drawn from LF as a marker of sympathetic modulation in this study should be interpreted with caution. Third, previous studies have showed that physical activity and inflammation could influence HRV [41, 45, 46]. The present study did not assess the contribution of physical activity levels or inflammation to the differences in HRV changes among individuals with neuropathic DFU or their counterparts without DFU. Due to the presence of ulcers or deformities, individuals with DFU need to reduce weight-bearing activity for offloading during hospitalization. Individuals with T2DM but without DFU usually engaged in less exercise and limited movement in the wards due to medical activities such as examination, treatment, and consultation during hospitalization (about 7–10 days). Thus, physical activity levels of these patients were low during Holter ECG monitoring. However, our research did not quantify the physical activity levels of the two groups and did not assess the impact of differences in physical activity on HRV. However, we performed 24-h ECG Holter monitoring for individuals with DFU at the proliferative phase of granulation tissue. At this phase, the inflammatory response is mild which could reduce the impact on HRV. Fourth, this study only suggested that cardiac autonomic regulation was more severely impaired in participants with neuropathic DFU than in their counterparts without DFU. However, the severity of impairment which could significantly increase cardiovascular mortality is not available. We will further uncover the answer in a prospective follow-up study. Finally, this study is a single-center study, which may be subject to selection bias. Therefore, further validation is needed in multicenter studies. In the present study, however, we applied a variety of measures to control confounding factors and achieve the comparability of baseline variables between two groups. Thus, the variation of these results should be small even if validation in multicenter studies.

## 5. Conclusions

We found that the regulation function of cardiac autonomic nerve was more severely impaired in people with neuropathic DFU than in their counterparts without DFU even if of similar age, diabetic duration, and DPN occurrence. The former has greater cardiac sympathovagal imbalance and lower vagal activity. Thus, in a manner of speaking, people with DM who are prone to developing DFU are more likely to have relatively severe abnormalities of cardiac autonomic modulation, even if of similar age, diabetic duration, and DPN. Further prospective studies are needed to verify whether the different severity of impairment could be responsible for the discrepancy of the risk of cardiovascular death between people with DFU and those with diabetes who have no history of foot ulceration.

## Figures and Tables

**Table 1:** Baseline demographic and clinical characteristics of all participants after PSM.

**Variables**	**DFU group**	**Non-DFU group**	**t**/**z**/**χ**^2^	**p**
No. of patients (**n**)	181	181		
Sex (men) (%)	117 (64.6)	122 (67.4)	0.308	0.579
Age (year)	59.8 ± 11.5	58.4 ± 13.0	−1.09	0.274
Diabetic duration (year)	12 (6, 17)	12 (6, 18)	−0.350	0.723
Alcohol consumption (%)			0.022	0.989
Never	114 (63.0)	113 (62.4)		
Occasional	28 (15.5)	29 (16.0)		
Frequent	39 (21.5)	39 (21.6)		
Current smoking (%)	78 (43.1)	84 (46.4)	0.402	0.526
BMI (kg/m^2^)	23.9 ± 3.7	23.8 ± 3.9	−0.28	0.780
SBP (mmHg)	138.0 ± 22.6	132.4 ± 19.9	−2.49	0.013
DBP (mmHg)	82.3 ± 13.4	82.7 ± 12.6	0.31	0.756
HbA1c (%)	8.4 (7.0, 9.8)	8.3 (7.0, 10.0)	0.153	0.879
FPG (mmol/L)	8.5 (6.5, 11.8)	7.8 (6.1, 11.6)	1.439	0.150
TC (mmol/L)	3.90 ± 1.08	3.92 ± 1.06	0.19	0.851
TG (mmol/L)	1.26 (1.01, 1.94)	1.27 (0.90, 1.95)	0.569	0.570
HDL-C (mmol/L)	1.07 ± 0.34	1.15 ± 0.43	1.98	0.048
LDL-C (mmol/L)	2.18 ± 0.87	2.15 ± 0.87	−0.35	0.724
eGFR (mL/min/1.73m^2^)	86.6 ± 22.6	87.9 ± 23.2	0.51	0.611
DPN	181 (100)	181 (100)	0.000	1.000
HBP	93 (51.4)	87 (48.1)	0.398	0.528
CVD	9 (5.0)	9 (5.0)	0.000	1.000
CAD	18 (9.9)	19 (10.5)	0.030	0.862

*Note:* Data are mean (SD), median (IQR), or *n* (percentage). Data analysis after PSM, with adjustment variables including age, sex, diabetic duration, BMI, MBP, smoking history, drinking history, family history of DM, HBP, CAD, CVD, DPN, eGFR, TC, TG, and HbA1C. HbA1c, glycated hemoglobin; TG, triglycerides; HBP, hypertension.

Abbreviations: BMI, body mass index; CAD, coronary artery disease; CVD, cerebrovascular disease; DBP, diastolic blood pressure; DFU, diabetic foot ulceration; DM, diabetes mellitus; DPN, diabetic peripheral neuropathy; eGFR, estimated glomerular filtration rate; FPG, fasting plasma glucose; HDL-C, high-density lipoprotein cholesterol; LDL-C, low-density lipoprotein cholesterol; SBP, systolic blood pressure; TC, total cholesterol.

**Table 2:** Indices of heart rate variability in participants with and without diabetic foot ulceration.

**Variables**	**DFU group**	**Non-DFU group**	**t**/**z**/**χ**^2^	**p**
No. of patients (**n**)	181	181		
HR (bpm)	80.6 ± 9.7	76.6 ± 11.2	−3.65	< 0.001
SDNN (ms)	64 (51, 86)	94 (71, 118)	−7.375	< 0.001
SDANN (ms)	57 (46, 80)	84 (63, 107)	−7.200	< 0.001
PNN50 (%)	0.3 (0.0, 2.5)	1.3 (0.2, 4.7)	−3.769	< 0.001
rMSSD (ms)	12 (10, 20)	18 (13, 23)	−4.570	< 0.001
HF (ms^2^)	4.51 (3.18, 7.51)	6.59 (4.80, 9.41)	−5.081	< 0.001
LF (ms^2^)	4.82 (2.76, 8.89)	10.27 (6.73, 14.52)	−8.037	< 0.001
LF/HF	1.02 (0.76, 1.42)	1.45 (1.19, 1.80)	−7.832	< 0.001

*Note:* Data are mean (SD), median (IQR), or *n* (percentage). Data analysis after PSM, with adjustment variables including age, sex, diabetic duration, BMI, MBP, smoking history, drinking history, family history of DM, HBP, CAD, CVD, DPN, eGFR, TC, TG, and HbA1C. SDNN, the standard deviation of normal sinus interval; SDANN, the standard deviation of the 5-min average RR intervals; PNN50, the percentage of normal adjacent RR interval difference > 50 ms; rMSSD, the root mean square of successive RR interval differences; HF, high-frequency power; LF, low-frequency power; LF/HF, rate of low-frequency power between high-frequency power.

Abbreviations: DFU, diabetic foot ulceration; HR, heart rate.

**Table 3:** The association of diabetic foot ulceration and indices of heart rate variability among participants with T2DM.

	**Univariate**		**Multivariable** ^**a**^	
	**β** **, 95% CI**	**p** ** value**	**β** **, 95% CI**	**p** ** value**
SDNN	−0.334 (−0.418, −0.251)	< 0.001	−0.331 (−0.412, −0.251)	< 0.001
LF/HF	−0.178 (−0.219, −0.137)	< 0.001	−0.174 (−0.213, −0.135)	< 0.001
SDANN	−0.321 (−0.404, −0.238)	< 0.001	−0.319 (−0.399, −0.238)	< 0.001
LF	−0.650 (−0.794, −0.506)	< 0.001	−0.651 (−0.786, −0.516)	< 0.001
PNN50	−0.238 (−0.421, −0.056)	0.011	−0.264 (−0.441, −0.088)	0.003
rMSSD	−0.220 (−0.316, −0.124)	< 0.001	−0.232 (−0.324, −0.140)	< 0.001
HF	−0.302 (−0.413, −0.191)	< 0.001	−0.310 (−0.418, −0.203)	< 0.001

*Note:* Data analysis after PSM, with adjustment variables including age, sex, diabetic duration, BMI, MBP, smoking history, drinking history, family history of DM, HBP, CAD, CVD, DPN, eGFR, TC, TG, and HbA1C. SDNN, the standard deviation of normal sinus interval; LF/HF, rate of low-frequency power between high-frequency power; SDANN, the standard deviation of the 5-min average RR intervals; LF, low-frequency power; PNN50, the percentage of normal adjacent RR interval difference > 50 ms; rMSSD, the root mean square of successive RR interval differences; HF, high-frequency power; HBP, hypertension; TG, triglycerides; HbA1c, glycated hemoglobin.

Abbreviations: BMI, body mass index; CAD, coronary artery disease; CVD, cerebrovascular disease; DM, diabetes mellitus; DPN, diabetic peripheral neuropathy; eGFR, estimated glomerular filtration rate; MBP, mean blood pressure; PSM, propensity score matching; T2DM, Type 2 diabetes mellitus; TC, total cholesterol.

^a^After PSM and then adjustment for variables, including age, sex, diabetic duration, BMI, MBP, smoking history, drinking history, family history of DM, HBP, CAD, CVD, eGFR, TC, TG, and HbA1C.

**Table 4:** The odds ratios (ORs) and 95% confidence intervals (95% CIs) of cardiac autonomic modulation impairment among participants with Type 2 diabetes mellitus, according to diabetic foot ulceration status.

**Analysis**	**Cardiac autonomic modulation impairment**
Multivariate analysis^a^	5.170 (3.026, 8.836), < 0.001

*Note:* Data analysis after PSM, with adjustment variables including age, sex, diabetic duration, BMI, MBP, smoking history, drinking history, family history of DM, HBP, CAD, CVD, DPN, eGFR, TC, TG, and HbA1C. HBP, hypertension; TG, triglycerides; HbA1c, glycated hemoglobin.

Abbreviations: BMI, body mass index; CAD, coronary artery disease; CVD, cerebrovascular disease; DM, diabetes mellitus; DPN, diabetic peripheral neuropathy; eGFR, estimated glomerular filtration rate; MBP, mean blood pressure; TC, total cholesterol.

^a^After PSM and then adjustment for variables, including age, sex, diabetic duration, BMI, MBP, smoking history, drinking history, family history of DM, HBP, CAD, CVD, eGFR, TC, TG, and HbA1C.

## Data Availability

Data that support the findings of this study are available from the corresponding author upon reasonable request.
